# Phylogenetic Placement and Phylogeography of Large-Flowered *Lotus* Species (Leguminosae) Formerly Classified in *Dorycnium*: Evidence of Pre-Pleistocene Differentiation of Western and Eastern Intraspecific Groups

**DOI:** 10.3390/plants10020260

**Published:** 2021-01-28

**Authors:** Tatiana E. Kramina, Maya V. Lysova, Tahir H. Samigullin, Ivan A. Schanzer, Mehmet U. Özbek, Dmitry D. Sokoloff

**Affiliations:** 1 Department of Higher Plants, Biological Faculty, Lomonosov Moscow State University, GSP-1, Leninskie Gory, 119234 Moscow, Russia; dmitry.sokoloff@msu-botany.ru; 2 LLC “Amplitech”, 1-ya Kuryanovskaya Str., 34-8, 109235 Moscow, Russia; m.lysova@amplitech.ru; 3 A.N. Belozersky Institute of Physico-Chemical Biology, Lomonosov Moscow State University, GSP-1, Leninskie Gory, 119991 Moscow, Russia; samigul@belozersky.msu.ru; 4 Tsitsin Main Botanical Garden of Russian Academy of Sciences, Botanicheskaya Str., 4, 127276 Moscow, Russia; ischanzer@gbsad.ru; 5 Department of Biology, Faculty of Science, Gazi University, 06500 Teknikokullar, Ankara, Turkey; ufukozbek@gazi.edu.tr

**Keywords:** *Lotus* sect. *Bonjeanea*, *Lotus strictus*, *Lotus rectus*, *Lotus hirsutus*, *Lotus graecus*, nrITS, *rps*16, *trn*L-F, Mediterranean, Messinian salinity crisis

## Abstract

The Mediterranean region is a center of species and genetic diversity of many plant groups, which served as a source of recolonization of temperate regions of Eurasia in Holocene. We investigate the evolutionary history of species currently classified in *Lotus* sect. *Bonjeanea* in the context of the evolution of the genus *Lotus* as a whole, using phylogenetic, phylogeographic and dating analyses. Of three species of the section, *L. rectus* and *L. hirsutus* have wide Mediterranean distribution while *L. strictus* has a disjunctive range in Bulgaria, Turkey, Armenia, Eastern Kazakhstan, and adjacent parts of Russia and China. We used entire nuclear ribosomal ITS1-5.8S-ITS2 region (nrITS) and a plastid dataset (*rps*16 and *trn*L-F) to reconstruct phylogenetic relationships within *Lotus* with an extended representation of *Bonjeanea* group. We analyzed the phylogeographic patterns within each species based on the plastid dataset. For divergence time estimation, the nrITS dataset was analyzed. Our results confirmed the non-monophyletic nature of the section *Bonjeanea*. They indicate that *Lotus* is likely to have diverged about 15.87 (9.99–19.81) million years ago (Ma), which is much older than an earlier estimate of ca. 5.54 Ma. Estimated divergence ages within *L. strictus*, *L. rectus*, and *L. hisrutus* (6.1, 4.94, and 4.16 Ma, respectively) well predate the onset of the current type of Mediterranean climate. Our data suggest that relatively ancient geological events and/or climatic changes apparently played roles in early diversification of *Lotus* and its major clades, as well as in formation of phylogeographic patterns, in at least some species.

## 1. Introduction

*Lotus* L. (incl. *Dorycnium* Mill.) is the largest genus of the tribe Loteae (Leguminosae-Papilionoideae) containing ca. 130 species of annual and perennial herbs, semishrubs, and shrubs or dwarf shrubs widely distributed in Eurasia, Africa, Australia and several islands of the Atlantic, Indian, and Pacific oceans [1]. Kramina and Sokoloff [2] and Sokoloff [3] divided *Lotus* into 14 sections, and this classification was used as a base for molecular phylogenetic studies [4,5,6,7]. In the phylogeny of the genus *Lotus*, whose major center of species diversity is located in the Mediterranean region, an early split into “southern” and “northern” evolutionary lineages was discovered [6]. The northern lineage is supported by plastid data only and includes members of four sections: sect. *Lotus*, sect. *Erythrolotus* Brand, sect. *Dorycnium* (Mill.) D.D. Sokoloff, and sect. *Bonjeanea* (Rchb.) D.D.Sokoloff [6]. The southern evolutionary lineage of the genus *Lotus* comprises members of all remaining currently recognized sections of the genus and can be distinguished using plastid, as well as nuclear markers (with the only exception concerning the position of *Lotus* sect. *Chamaelotus* Kramina et D.D. Sokoloff) [6]. Previous phylogenetic studies of *Lotus* [4,5,6,8] revealed many conflicts between taxonomic systems based on morphology and phylogenetic tree topologies obtained using different DNA markers. One of the problems in the taxonomy of the genus *Lotus* is its separation from closely related genera [1,3,6,8,9]. *Lotus* and *Dorycnium* were consistently distinguished as two distinct genera in European “Floras” in pre-molecular times (e.g., References [10,11]), though this concept was criticized in accounts considering New World material [9,12]. Meanwhile, several species occupy unstable taxonomic positions between these genera, which led to their transfer from *Lotus* to *Dorycnium*, or vice versa, and sometimes to recognition as independent genera. The first group of species with such uncertain position is associated with the taxon “*Bonjeanea*”. Reichenbach [13] segregated a new genus, *Bonjeanea* Rchb. as having a habit intermediate between those of *Lotus* and *Dorycnium*, with two species, *Bonjeanea recta* (L.) Rchb. and *B. hirsuta* (L.) Rchb. Rikli [14] monographed *Dorycnium* and subdivided the genus into three sections: *Canaria* Rikli, *Bonjeanea* (Rchb.) Taubert, and (*Eu*)*Dorycnium.* He included three species in the section *Bonjeanea*: *D. rectum* (L.) Ser.*, D. hirsutum* (L.) Ser., and *D. latifolium* Willd. (=*D. graecum* (L.) Ser.). Lassen [15] noted the morphological similarity between *D. hirsutum* and *Lotus strictus* Fisch. et C.A.Mey. and transferred the latter to the genus *Dorycnium*. On the basis of morphological data, Gillett [16] excluded the section *Canaria* from the genus *Dorycnium* and transferred it to the genus *Lotus* as a subgenus. Later, its rank was reduced to sectional [3].

Sokoloff [17] formulated a syndrome of characters of the “core *Dorycnium*”, which included *D. pentaphyllum* Scop. s.l. and related species, *D. sanguineum* Vural, *D. axilliflorum* Hub.-Mor., and *D. fulgurans* (Porta) Lassen, as well as *D. graecum* (Table 1). Having compared morphological and molecular phylogenetic data, Sokoloff [3] followed Polhill [9] in lumping genera *Lotus*, *Dorycnium*, and *Bonjeanea*. In his treatment, *Lotus* section *Dorycnium* (Mill.) D.D. Sokoloff corresponded to the “core *Dorycnium*”, *Lotus* section *Bonjeanea* (Rchb.) D.D. Sokoloff corresponded to the genus *Bonjeanea* [13] plus *L. strictus*, and *Lotus* section *Canaria* (Rikli) D.D. Sokoloff corresponded to the *Dorycnium* section *Canaria*.

Current classification of Leguminosae is largely based on phylogenetic analyses of plastid markers [18]. Within Papilionoideae, *Lotus* belongs to the “50-kb inversion clade”, whose members have a 50-kb inversion in the large single-copy region of the plastome. Within the “50-kb inversion clade”, *Lotus* belongs to the NPAAA (non-protein amino acid-accumulating) clade that includes the majority of agriculturally cultivated legumes [19]. Molecular phylogenetic analyses of plastid *trn*K-*mat*K sequences clearly demonstrated that *Lotus* and related genera (i.e., tribe Loteae) belong to the Robinioid clade. *Sesbania* Adans., a genus from palaeo- and neotropics, is a sister group of the tribe Loteae, and Robinieae (=robinioid crown clade), which includes several genera of trees, shrubs and sometimes herbs from tropics and subtropics of the New World_­_, is a sister group to the clade of Loteae plus *Sesbania* [19,20,21,22].

An important progress has been achieved in dating main evolutionary events in the phylogeny of legumes. The family Leguminosae diversified during the Early Tertiary. First definitely determined legumes appeared in the Late Paleocene, about 56 Ma [23]. The legume stem clade age was estimated at 59.9 Ma [20]. The Papilionoideae stem clade age was set to a minimum of 55 Ma (Late Paleocene) on the basis of fossil records. The robinioid crown clade age, i.е., the MRCA (the most recent common ancestor) of *Hebestigma* Urb. and *Robinia* L., was evaluated as 45.4 Ma or 48.3 Ma, according to *rbc*L and *mat*K based phylogenetic reconstructions, respectively [20]. The *Robinia* stem clade was directly dated at a minimum of 33.7 Ma based on the fossil wood of *Robinia zirkelii* (Platen) Matten, Gastaldo & Lee [24,25,26]. The age of the clades Loteae-*Sesbania* and Loteae was estimated on the basis of previous dating analyses as 36.7 ± 1.8 and 21.5–24.6 Ma, respectively [20,22]. Based on the dating of Lavin et al. [20], Jaén-Molina et al. [7] estimated the time when *Lotus* separated from its sister group (i.e., *Hammatolobium* Fenzl and *Cytisopsis* Jaub. & Spach) at 7.86 Ma, and the time of diversification of extant clades of the genus at 5.54 Ma.

The main center of species diversity of the genus *Lotus* is located in the Mediterranean region [6]. There is also another important center in Macaronesia, but it is presumably much younger than the Mediterranean one [7]. In recent decades, phylogeography of Mediterranean plants has been extensively studied, most of the studies being focused on sclerophytes and woody plants typical for vegetation of this region (reviewed by References [27,28,29]). The vast species richness of the Mediterranean basin, one of the world biodiversity hotspots [30,31,32], is explained by biogeographic patterns associated with highly heterogeneous landscape, complex geological and climatic history, and long-term human activity [29,33]. The three large Mediterranean peninsulas, i.e., Iberian, Apennine, and Balkan, as well as Anatolia, accumulate much genetic and species diversity, which generally decreases towards higher latitudes. The peninsulas acted as refugia for many species through Pleistocene climatic oscillations [28]. The Mediterranean area was less affected by the latest glaciations [27], and the genetic structure of some Mediterranean species may be the result of older processes (e.g., Reference [33]). Presumably existing from the Tertiary period, Mediterranean refugia are climatically stable areas for long-term conservation of species and genetic diversity [27]. They served as sources for recolonization of central and northern Europe during interglacials [28].

In this connection, the biogeography of the northern evolutionary lineage of *Lotus* is of particular interest, since each of the sections of the northern lineage includes species common in the Mediterranean, as well as species that have advanced farthest into temperate latitudes. In this paper, we investigate the evolutionary history of three species of *Lotus* sect. *Bonjeanea* in the context of the evolution of the genus *Lotus* as a whole, using phylogenetic, phylogeographic and dating analyses. Two currently recognized species of *Lotus* sect. *Bonjeanea* (*L. hirsutus* L. and *L. rectus* L.) have wide Mediterranean ranges, and the third species (*L. strictus*) is spread from east Mediterranean to south-western Siberia and Northern Dzungaria. We also study phylogeography of *L. graecus* L., formerly included in the section *Bonjeanea*, as well as related Turkish endemic species *L. axilliflorus* (Hub.-Mor.) D.D. Sokoloff and *L. sanguineus* (Vural) D.D. Sokoloff. Sampling of species for the present study is largely dictated by their conflicting positions in earlier analyses of morphological, nuclear and plastid data [4,6,17]. Basically, we deal with all species placed in *Dorycnium* by Lassen [15] and Greuter et al. [34] except members of the highly polymorphic complex of *Lotus dorycnium* sensu lato. The *L. dorycnium* complex includes a few small-flowered species whose limits, diagnostic characters, and phylogeographic patterns will be discussed separately.

Our objectives are: (1) to detail genetic diversity in the species of the *Bonjeanea* group (i.e., *Lotus rectus*, *L. strictus*, *L. hirsutus*, and *L. graecus*) across the distribution ranges using molecular data and phylogenetic analyses of the genus *Lotus* with extended representation of the *Bonjeanea* group; (2) to assess the phylogeographic patterns and infer the main drivers of differentiation among studied species*;* and (3) to estimate the ages of major subclades of the genus *Lotus* using molecular phylogenetic analyses.

## 2. Results

### 2.1. Analysis of nrDNA ITS

The ITS1-5.8S-ITS2 region (nrITS) dataset included 107 accessions (Supplementary Materials, Dataset S1), 104 in the ingroup (i.e., *Lotus*) and three in the outgroups (i.e., *Hammatolobium kremerianum, Cytisopsis lotoides* and *Tripodion tetraphyllum*). One hundred percent identical sequences of the same species were combined into one accession. The ingroup covers 31 species of *Lotus* and 13 of 14 sections of the genus with enlarged representation of *Lotus rectus*, *L. hirsutus*, *L. strictus* and *L. graecus*. The total alignment length was 667 bp (617 bp after the exclusion of gap-rich and ambiguous positions).

**Figure 1:**
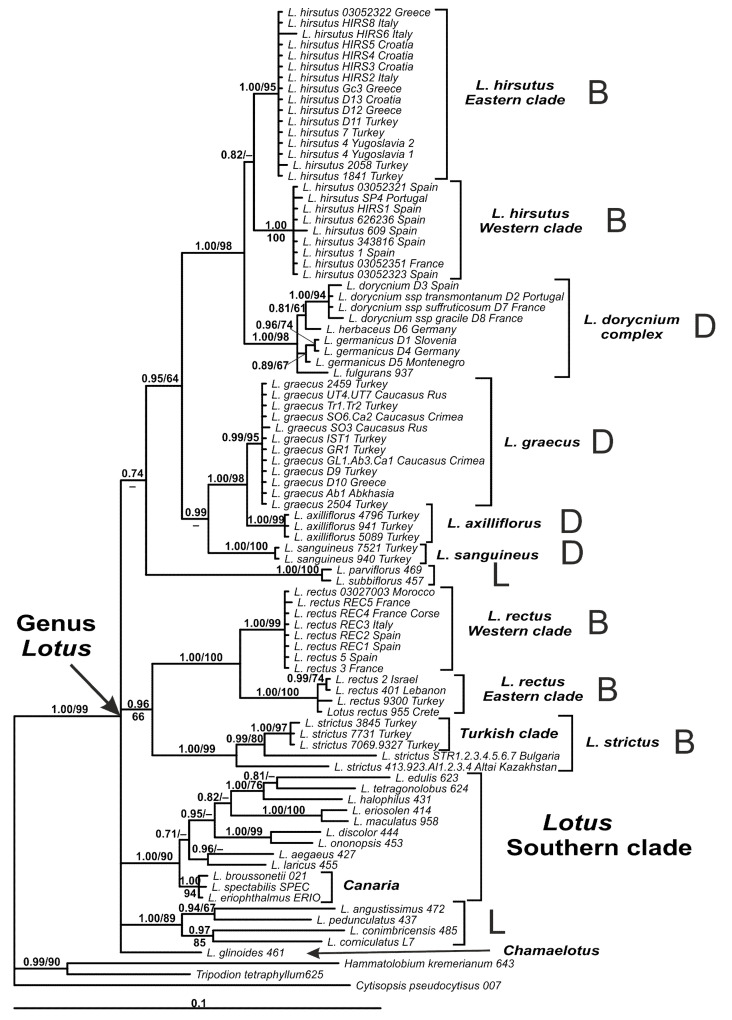
Phylogenetic relationships in *Lotus* inferred from Bayesian analysis of the ITS1-5.8S-ITS2 region (nrITS) dataset. Branch length is proportional to the number of expected nucleotide substitutions, scale bar corresponds to 0.1 substitutions per site. Numbers above branches are posterior probabilities. Numbers below branches or after slashes are bootstrap support values found in Maximum Likelihood analysis of the same dataset (values equal or more than 0.6/60% shown). Sections of the Northern evolutionary lineages of *Lotus* (according to Reference [3]) are marked with letters: B–*Bonjeanea*, D–*Dorycnium*, L–*Lotus* (incl. *Erythrolotus*). Sample codes and country of origin for sequences are given after species names. See Appendix A for voucher information.

The genus *Lotus* clade is well supported by Bayesian and maximum likelihood (ML) analyses (posterior probabiliy PP 1.00, bootstrap support BS 99%) and further splits into several branches (Figure 1). One of them is the *Lotus* Southern clade (PP 1.00, BS 89%), which contains members of eight *Lotus* sections (i.e., *Krokeria* (Moench) Ser., *Tetragonolobus* (Scop.) Benth. et Hook. f., *Lotea* (Medik.) DC.*, Pedrosia* (Lowe) R.P. Murray*, Rhyncholotus* (Monod) D.D. Sokoloff, *Heinekenia* Webb et Berth., *Ononidium* Boiss., and *Canaria*), but does not include *L. glinoides* Delile, which is the only member of *Lotus* sect. *Chamaelotus* sampled here. Members of *Lotus* sect. *Lotus* are represented by two unrelated clades, marked with the letter L on the tree (i.e., *L. parviflorus* Desf. group and a clade of remaining species of the section). Other clades represent *Lotus* sections *Dorycnium* (marked with D) and *Bonjeanea* (marked with B). Both sections are not monophyletic. *Lotus* section *Bonjeanea* is represented by a clade of *L. rectus* plus *L. strictus* (PP 0.96, BS 66%) and a clade of *L. hirsutus*, which is weakly supported by Bayesian analysis only (PP 0.82). Species of *Lotus* section *Dorycnium* are grouped into two clades, *L. dorycnium* complex clade (PP 1.00, BS 98%) and a clade of *L. graecus* and related species *L. axilliflorus* and *L. sanguineus*, supported by Bayesian analysis only (PP 0.99).

**Figure 2:**
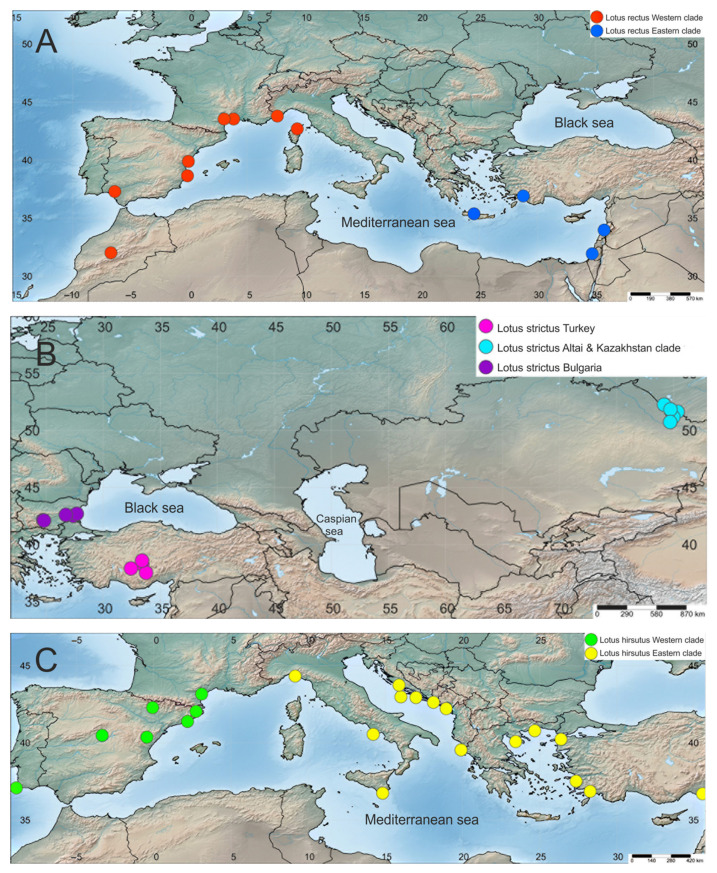
Geographical distribution of clades revealed in nrITS phylogenetic analysis of species: (**A**)—*L. rectus*, (**B**)—*L. strictus*, (**C**)—*L. hirsutus*.

Both Bayesian and ML analyses strongly support the separation of *L. rectus* into Western (PP 1.00, BS 99%) and Eastern (PP 1.00, BS 100%) subclades (Figure 1 and Figure 2A). A similar pattern of clusterization is observed in *L. hirsutus*. It also splits into Western (PP 1.00, BS 100%) and Eastern (PP 1.00, BS 95%) branches, which are however shorter than the corresponding branches of *L. rectus* (Figure 1 and Figure 2C). Turkish specimens of *L. strictus* form a clade (PP 1.00, BS 97%) which is consistently combined first with the Bulgarian, and then with the Altai-Kazakhstan samples (Figure 1 and Figure 2B). *Lotus graecus* forms a well-supported clade with *L. axilliflorus*, but *L. sanguineus* is more distantly related. Bayesian and ML analyses undoubtedly classified three species of *L*. sect. *Canaria* (i.e., *L. broussonetii* Choisy ex Ser., *L. spectabilis* Choisy ex Ser., and *L. eriophthalmus* Webb et Berth.) within the *Lotus* Southern clade and did not support their close relationships with any member of the former genus *Dorycnium*.

### 2.2. Analysis of Plastid DNA Dataset

The plastid DNA dataset included 107 sequences representing the same outgroup and ingroup as in nrITS analysis plus the sequence of *L. hirsutus* 03052324 (Supplementary Materials, Dataset S2). The total alignment length of the combined dataset was 1838 bp (incl. *rps*16 intron of 891 bp and *trn*L-F of 947 bp), but, after the exclusion of gap-rich and ambiguous positions, the alignment length was reduced to 1703 bp.

**Figure 3:**
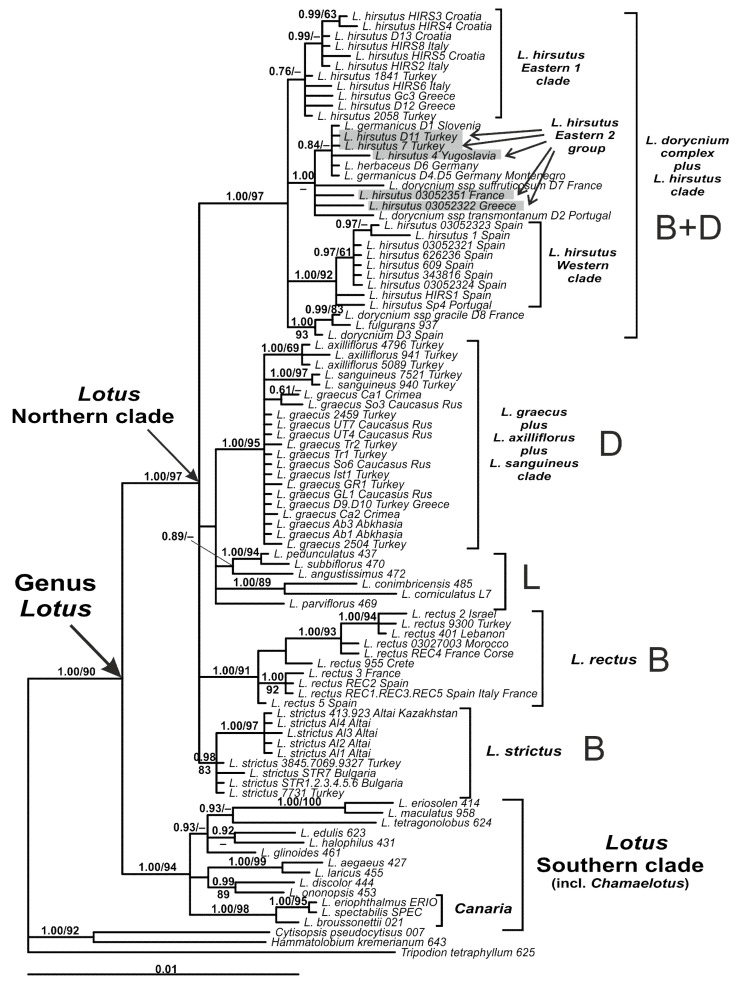
Phylogenetic relationships in *Lotus* inferred from Bayesian analysis of the plastid DNA dataset. Branch length is proportional to the number of expected nucleotide substitutions, scale bar corresponds to 0.01 substitutions per site. Numbers above branches are posterior probabilities. Numbers below branches or after slashes are bootstrap support values found in Maximum Likelihood analysis of the same dataset (values equal or more than 0.6/60% shown). Sections of the Northern evolutionary lineages of *Lotus* are marked as on Figure 1. Sample codes and country of origin for sequences are given after species names. See Appendix A for voucher information.

The genus *Lotus* is highly supported by Bayesian phylogenetic analysis (PP 1.00) and slightly weaker by ML analysis (BS 90%) (Figure 3). Both analyses demonstrate the split of *Lotus* into the Northern (PP 1.00, BS 97%) and Southern (PP 1.00, BS 94%) clades. The Southern clade combines *Lotus* species, which belong to nine sections, including *L*. sect. *Canaria* and *Chamaelotus*. The *Lotus* Northern clade includes several rather well supported branches, i.e., *L. dorycnium* complex plus *L. hirsutus* clade, *L. graecus* plus related species clade, two separate clades and a branch of *L. parviflorus* of *L*. sect. *Lotus*, *L. rectus* clade and *L. strictus* clade. Monophyly of none of the sections of the Northern clade (i.e., *L*. sect. *Lotus*, *Dorycnium*, and *Bonjeanea*) was confirmed by both methods of analysis.

The analysis of the plastid dataset revealed a well-supported clade comprising all accessions of *L. graecus*, *L. axilliflorus*, and *L. sanguineus*, but failed to support monophyly of *L. graecus*. Both *L. axilliflorus* and *L. sanguineus* are revealed as well-supported clades on relatively long branches.

The clade of *L. rectus* includes a basal grade of samples from the western part of its range, an intermediate grade of specimens from Morocco and Mediterranean islands (Crete and Corse) and an Eastern clade. The clade of *L. strictus* includes a basal grade formed by Turkish and Bulgarian samples and a clade of Altai-Kazakhstan specimens.

Within the clade (*L. dorycnium* complex plus *L. hirsutus*), the sequences of *L. hirsutus* are included in three subclades: some of them form a fairly well supported Western subclade (PP 1.00, BS 92%), the other part is combined in a less supported Eastern 1 subclade (PP 0.76), and the third part (Eastern 2 group) is intermingled with members of *L. dorycnium* complex in a common subclade.

Similar to nrITS analysis, the shorter branches were observed: in Turkish, rather than in Altai-Kazakhstan, samples of *L. strictus*; in western, rather than in eastern, samples of *L. rectus*; in eastern, rather than in some western, samples of *L. hirsutus*.

### 2.3. Phylogeography Based on the Plastid Dataset

#### 2.3.1. *Lotus rectus*

**Figure 4:**
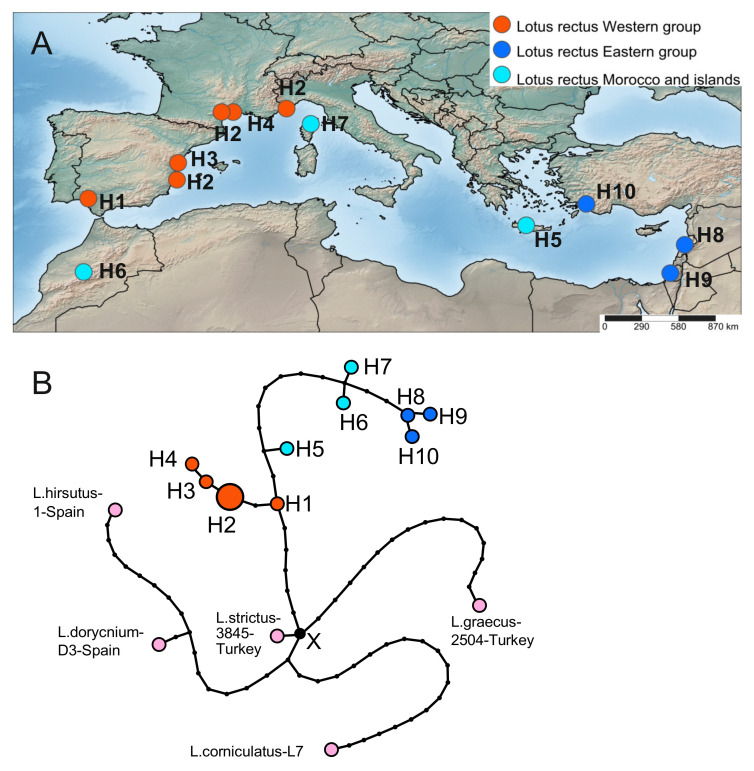
Geographical distribution of haplotypes identified in *L. rectus* (**A**) and haplotype network (**B**) reconstructed based on the combined plastid DNA dataset. The size of each circle is proportional to the frequency of the haplotype in the dataset. *L. strictus*, *L. hirsutus*, *L. dorycnium*, *L. graecus*, and *L. corniculatus* represent outgroups.

We analyzed 17 sequences, twelve in the ingroup (i.e., *L. rectus*) and five in the outgroup, represented by *L. strictus*, *L. hirsutus*, *L. dorycnium*, *L. graecus*, and *L. corniculatus* (Figure 4). The program calculated the parsimony limit of 30 steps and collapsed sequences into 15 haplotypes, five in the outgroup and ten in *L. rectus* (H1–H10). Haplotype diversity (Hd) and nucleotide diversity (pi) in *L. rectus* sequences are rather high (0.909 and 0.00436, respectively). On a parsimony network constructed using TCS software (Figure 4B), the internal haplotype H1 from Spain is the closest to the hypothetical haplotype X, which represents the connection point with sequences of the outgroups (*L. strictus*, *L. graecus*, and other species of *Lotus*). The haplotype H1 differs from X by six mutations. The haplotype H1 is further connected to a branch of haplotypes H2–H4, which differ from it by two, three and four mutations, respectively. The haplotypes H1–H4 are distributed in Spain, France, and north-western Italy and represent a western group of *L. rectus* (Figure 4A). A long series of mutations (12 or 13) connects the haplotype H1 with haplotypes H8–H10 from the *L. rectus* eastern group, which includes samples from Lebanon, Israel, and Turkey. The haplotypes H5, H6, and H7 occupy intermediate positions between western and eastern groups of haplotypes, being closer to the first (H5 from Crete) or second (H6 from Morocco and H7 from Corse). The distribution of pairwise differences between studied sequences is multimodal (Supplementary Figure S1A), which may indicate that *L. rectus* has been widespread in this area for a long time and has repeatedly expanded and reduced in number. The preliminary morphological analysis of herbarium specimens did not reveal clear morphological differences between western and eastern populations of *L. rectus*.

#### 2.3.2. *Lotus strictus*

**Figure 5:**
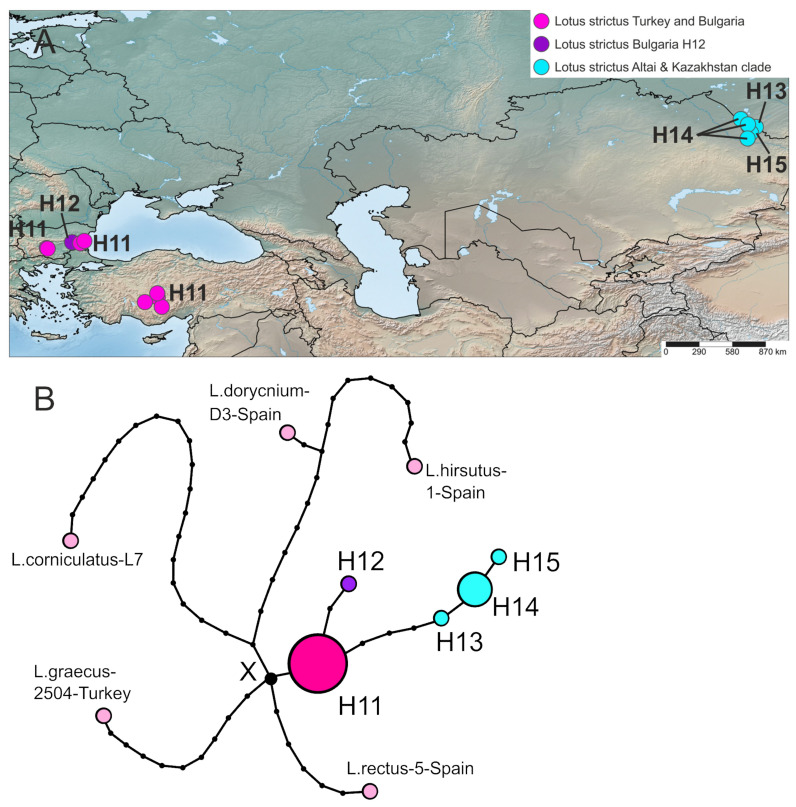
Geographical distribution of haplotypes identified in *L. strictus* (**A**) and haplotype network (**B**) reconstructed based on the combined plastid DNA dataset. The size of each circle is proportional to the frequency of the haplotype in the dataset. *L. rectus*, *L. hirsutus*, *L. dorycnium*, *L. graecus*, and *L. corniculatus* represent outgroups.

Of the 22 plastid sequences studied, 17 belonged to *L. strictus*, and five belonged to the outgroup, represented by *L. rectus*, *L. hirsutus*, *L. dorycnium*, *L. graecus*, and *L. corniculatus* (Figure 5). The program calculated the parsimony limit of 30 steps and collapsed sequences into 10 haplotypes, five in the outgroup and five in *L. strictus* (H11–H15). *L. strictus* is characterized by lower gene (Hd = 0.596) and nucleotide diversity (pi = 0.00141) compared to *L. rectus*. On a parsimony TCS network of *L. strictus* haplotypes (Figure 5), the haplotype H11 is the closest to the hypothetical haplotype X, differing from the latter by the only mutation. The haplotype H11 is the most frequent haplotype present in 10 samples from Turkey and Bulgaria. H11 is connected by a short branch of two mutations with the singleton H12 from Bulgaria. The other longer branch connects the haplotype H11 with a group of Altai-Kazakhstan haplotypes H13, H14, and H15, which are consecutively connected to each other and differ from H11 by four, five, and six mutations, respectively. Among this group of haplotypes, the haplotype H14 is more frequent (was found in four specimens). According to the distribution of pairwise differences (Supplementary Figure S1B), the species *L. strictus* also experienced fluctuations in population size and went through the bottlenecks. Now, it is not currently expanding. Studied herbarium specimens of *L. strictus* are variable by morphology, but our preliminary morphological analysis did not reveal clear morphological differences between Anatolian-Bulgarian and Altai-Kazakhstan populations.

#### 2.3.3. *Lotus hirsutus*

**Figure 6:**
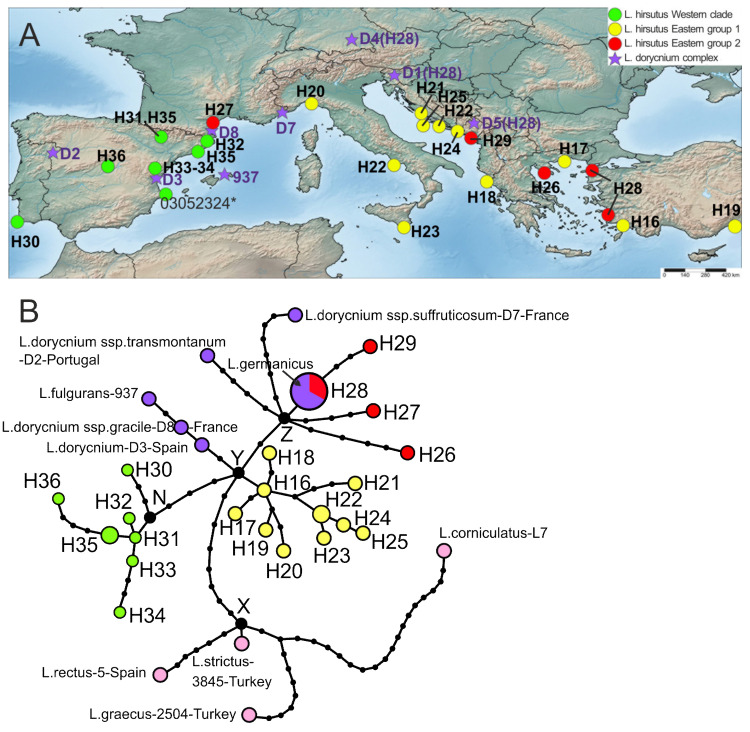
Geographical distribution of haplotypes identified in *L. hirsutus* (**A**) and haplotype network (**B**) reconstructed based on the combined plastid DNA dataset. The size of each circle is proportional to the frequency of the haplotype in the dataset. *L. dorycnium* complex, *L. strictus*, *L. rectus*, *L. graecus*, and *L. corniculatus* represent outgroups. The haplotype of *L. hirsutus* sample 03052324* was excluded from the network because of incomplete sequence *rps*16 intron.

We analyzed 37 sequences, including 24 in *L. hirsutus*, nine in *L. dorycnium* complex, and four in the outgroup, represented by *L. strictus*, *L. rectus*, *L. graecus*, and *L. corniculatus* (Figure 6). The program calculated the parsimony limit of 30 steps and collapsed sequences into 30 haplotypes, four in the outgroup, five in *L. dorycnium* complex, 20 in *L*. *hirsutus* (H16–H27, H29–H36), and one haplotype (H28) shared by *L. hirsutus* and *L. dorycnium* s.l. (Figure 6). The haplotype diversity within *L. hirsutus* (0.960) was the highest among all studied species, while nucleotide diversity (0.00392) was lower than that of *L. rectus*, but higher than in *L. strictus*. On a TCS network of haplotypes (Figure 6B), the hypothetical haplotype Y is a first putative center of divergence of *L. hirsutus*, which differs from the hypothetical haplotype X by eight mutations. Two main subclades of *L. hirsutus* (i.e., western subclade and eastern 1 subclade) may originate from the haplotype Y. The eastern 1 subclade includes haplotypes H16–H25 which are distributed from Turkey to Italy. This group of haplotypes starts to diverge from the haplotype H16 from Turkey which differs by the only mutation from the hypothetical haplotype Y. The eastern 1 sublclade of *L. hirsutus* is the most variable group by Hd (0.927) within the species. The western subclade includes haplotypes H30–H36 from the Iberian peninsula. It presumably starts to diverge from the hypothetical haplotype N, differing from the haplotype Y by four mutations. This group includes mainly singletons (except for H35), which are either internal, or tip by position.

Two other branches are connected to the point Y, these are the branch towards the third group of haplotypes, *L. hirsutus* eastern 2 group, and the branch of haplotypes of *L. dorycnium* complex. The eastern 2 group of *L. hirsutus* haplotypes, like several haplotypes of the *L. dorycnium* complex, possibly originates from the point Z, a hypothetical haplotype which differs from the haplotype Y by two mutations. The closest haplotype to Z is haplotype H28 shared by two Turkish specimens of *L. hirsutus* (samples 7 and D11), as well as three specimens of *L. germanicus* (samples D1, D4, and D5), a member of *L. dorycnium* complex. Samples D1, D4, and D5 are geographically located in S. Germany and in the Balkans. Three other haplotypes (H26, H27, and H29) from the Balkans and France also belong to the eastern 2 groups of *L. hirsutus.* They are connected to either Z point or the haplotype H28, differing by several mutations.

To summarize, the haplotypes of *L. hirsutus* can be divided into three groups, i.e., western, eastern 1 and eastern 2, and the eastern 2 group of haplotypes is combined with haplotypes of *L. dorycnium* complex, moreover, there is one haplotype (H28) shared by two taxa. The distribution of pairwise differences in a plastid set of *L. hirsutus* is multimodal, which indicates a long-term existence of the species in its distribution area and occurrence of demographic fluctuations (Supplementary Figure S1C).

#### 2.3.4. *Lotus graecus* and Related Taxa

**Figure 7:**
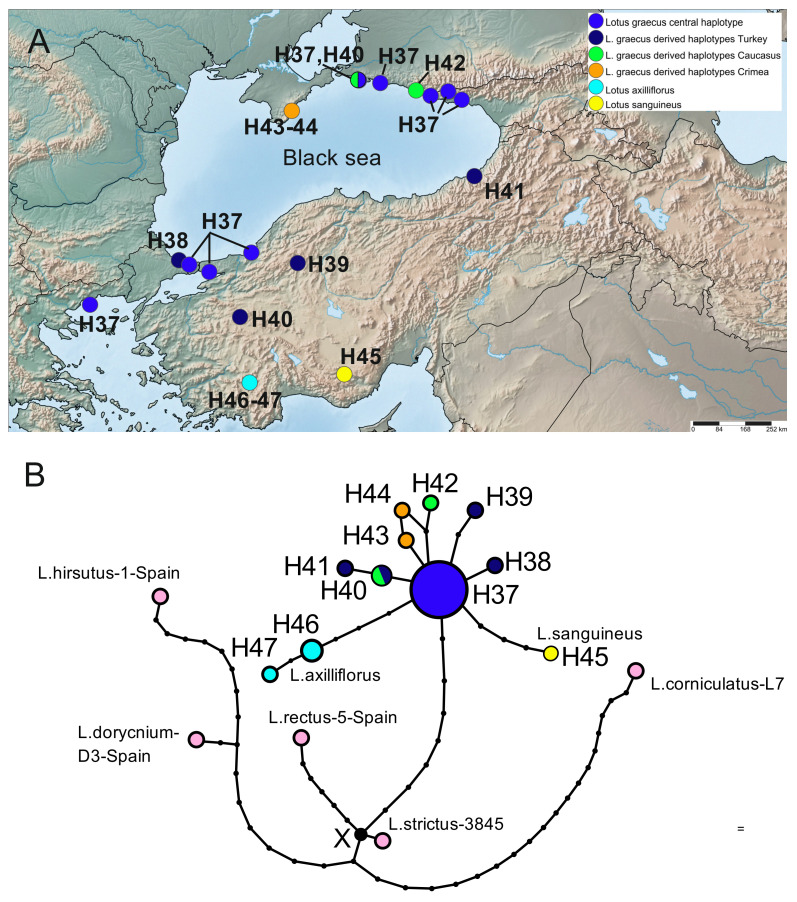
Geographical distribution of haplotypes identified in *L. graecus*, *L. axilliflorus*, and *L. sanguineus* (**A**) and haplotype network (**B**) reconstructed based on the combined plastid DNA dataset. The size of each circle is proportional to the frequency of the haplotype in the dataset. *L. rectus*, *L. hirsutus*, *L. strictus*, *L. dorycnium*, and *L. corniculatus* represent outgroups.

We studied 27 plastid sequences, including 17 sequences of *L. graecus*, three sequences of *L. axilliflorus*, two sequences of *L. sanguineus* and five sequences of the outgroup, represented by *L. rectus*, *L. hirsutus*, *L. strictus*, *L. dorycnium*, and *L. corniculatus*. The program calculated the parsimony limit of 30 steps and collapsed sequences into 16 haplotypes, eight in *L. graecus*, two in *L. axilliflorus*, one in *L. sanguineus*, and five in the outgroup (Figure 7). The haplotype diversity of *L. graecus* sequences is comparatively low and equal to that of *L. strictus* (Hd = 0.596), and its nucleotide diversity (0.00055) is the lowest among studied species. The central haplotype H37, widely distributed in Turkey, Greece and the Caucasus, is the most frequent (observed in 9 samples) (Figure 7). Other haplotypes (H38–H44) differ from it by one or two mutations and have narrow geographic distribution. They are mainly singletons except for H40, which was recorded in two samples from Turkey and the Caucasus. Two haplotypes derived from H37 were identified in two Crimean samples. The distribution of pairwise differences is unimodal with maximum at 0, which may indicate that this species has recently undergone demographic expansion, while low values of diversity coefficients may be evidence of its relatively small age (Supplementary Figure S1D).

The haplotypes of both Turkish endemic species (*L. sanguineus* and *L. axilliflorus*) seem to originate from the haplotype of *L. graecus*. Both samples of *L. sanguineus* belong to the same haplotype H45, which is related to the central haplotype of *L. graecus*, differing from the latter by four mutations. *L. axilliflorus* is characterized by two haplotypes, the internal haplotype H46 and the tip haplotype H47, which differ from the central haplotype of *L. graecus* by four and six mutations, respectively.

### 2.4. Dating Phylogeny of Lotus

**Figure 8:**
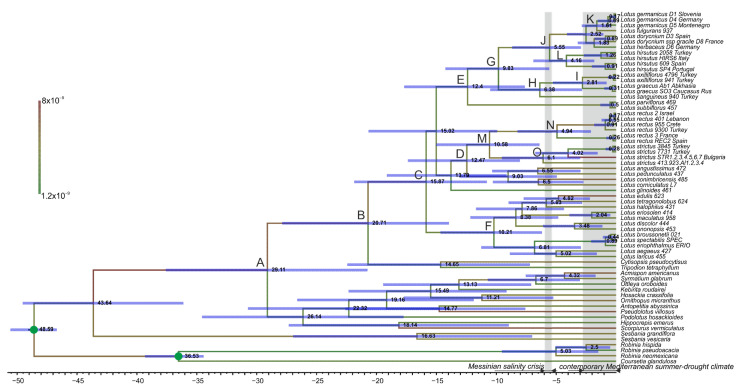
Chronogram summarizing results of the Bayesian dating analysis of nrITS dataset (74 accessions representing the genus *Lotus*, thirteen other genera of Loteae and *Sesbania*, *Robinia*, and *Coursetia* as outgroup). Mean age estimates in million years (Ma) are indicated for nodes, with node bars showing the associated 95% HPD credibility intervals. Age constraints were applied to nodes marked by green dots. Branch colors correspond to inferred mean substitution rates according to the scale bar. The major clades (**A**–**O**) are described in Table 2.

Results of Bayesian dating analyses with different speciation priors were very similar, so we present and discuss only results obtained using the Birth–Death branching pattern (Figure 8). The analyses revealed a medium level of rate heterogeneity with the mean of the coefficient of rate variation 0.53 (95% highest posterior density HPD interval 0.3744–0.6978) and mean clock rate 4.22 × 10^−9^ substitutions/site/year. The mean age of the Loteae-*Sesbania* clade is estimated from nrITS sequences at 43.64 Ma (95% HPD: 32.47–49.09 Ma), whereas the Loteae crown age is 29.11 (18.28–34.82) Ma (Figure 8, Table 2). Our results indicate that *Lotus* likely diverged about 15.87 (9.99–19.81) Ma. Within *Lotus*, the mean ages of large clades (i.e., *Lotus* Northern clades 1 and 2 and *Lotus* Southern clade) varied within 10.21–12.47 Ma, while species of *Lotus* section *Bonjeanea*, *L. strictus*, *L. rectus*, and *L. hisrutus,* likely start to diverge at mean ages estimated as 6.1, 4.94, and 4.16 Ma, respectively.

## 3. Discussion

### 3.1. Diversification of the Genus Lotus Much Pre-Dates Formation of the Extant Mediterranean Biome

Our data provide an updated framework for understanding tempo of evolution of the genus *Lotus*. Recently, Jaén-Molina et al. [7] published a dated phylogeny of *Lotus* based on nrITS sequences of 116 species, which is about 94% of the total species diversity in the genus [4], plus nine species from five closely related genera within Loteae and two species of *Sesbania* as outgroups. The analysis of Jaén-Molina et al. [7] was focused on colonization of Macaronesian islands, representing a collection of relatively recent diversification and dispersal events mostly confined to a particular clade (the *Pedrosia* clade). Our dating analysis is also based on nrITS sequences, but it is focused on several lineages that are closer to the root of *Lotus* than the *Pedrosia* clade. Therefore, our taxon sampling scheme involved a much broader set of outgroups that included most currently recognized genera of the tribe and major clades of *Sesbania* and Robinieae. While Jaén-Molina et al. [7] used an estimate age of Loteae inferred from an analysis of a *mat*K dataset [20], we found it reasonable to use the robinioid legumes age as a secondary calibration point, which is less-dependent on the analyzed dataset [20]. Jaén-Molina et al. [7] estimated an age of the crown group of *Lotus* at an average of 5.54 (3.45–7.90) Ma (i.e., close to the Pliocene to Miocene boundary), but our analysis revealed an about three times older the Miocene estimate. Consequently, ages of other clades within the genus are also older than those in the study of Jaén-Molina et al. [7].

As the genus *Lotus* and the tribe Loteae have major diversity centers in the Mediterranean, dated phylogenies have to be discussed in the context of important events in climate and geology of the Mediterranean region. The most important series of events of the Late Miocene is the Messinian salinity crisis (MSC) (5.96 to 5.33 Ma), when the Mediterranean Sea had no stable connection to the Atlantic Ocean and exhibited a great desiccation. This period ended with the appearance of the Gibraltar strait (the Zanclean flood) [35]. The Messinian salinity crisis played important roles in plant evolution and distribution patterns [28]. The influence of MSC on the formation of the diversity of Mediterranean plant lineages has been extensively discussed. In the study of *Stauracanthus* Link (Leguminosae), Pardo et al. [36] documented the negative effects of MSC causing plant retractions and local extinctions. The studies of Jabbour and Renner [37] on *Consolida* Gray (Ranunculaceae) and Lledó et al. [38] on *Limonium* Mill. (Plumbaginaceae)*,* on the opposite, demonstrated the role of MSC as a factor facilitating geographic expansion and promoting radiations. The data of Jaén-Molina et al. [7] suggest that major contemporary lineages of the genus (including the Northern and Southern groups [6]) appeared close to the Messinian/Zanclean border. In contrast, our analysis suggests that both Northern and Southern groups were well diversified by the beginning of the Messinian crisis. Moreover, our inferred mean crown group age of apparently the most enigmatic species of the genus, *L. strictus*, pre-dates the Messinian crisis and our estimates for two other members of the section *Bonjeanea*, *L. hirsutus* and *L. rectus*, are close to the time of the crisis, at least with respect to their stem groups. Importantly, our inferred crown clade ages of all three members of the section *Bonjeanea* exceed existing estimates of the age of the contemporary Mediterranean summer-drought regime (c. 2.8 Ma [39]).

Phylogeographic differentiation between western and eastern parts of distribution ranges is documented in several Mediterranean [29,40,41,42,43] and submiditerranean [44] angiosperm species. Apparently, such differentiation did not appear synchronously in various taxa. Our data suggest that differentiation between western and eastern lineages of *L. hirsutus* and *L. rectus* most likely took place as early as in Pliocene. In contrast, a similar differentiation in another member of the tribe Loteae, the submediterranean species *Anthyllis montana* L. has been dated as Late Quaternary [44]. Despite the apparently much younger age of geographical separation, morphological differentiation between the western and eastern groups of *A. montana* [45] is much more pronounced than between the groups within *L. rectus* and *L. hirsutus*. The very limited incongruence between molecular and morphological patterns of differentiation in *A. montana* somewhat resembles the incongruence between the plastid and molecular marker we observed in *L. hirsutus* and suggest complex evolutionary histories of these lineages. Kropf et al. [44] revealed a western Mediterranean origin followed by an eastward migration in *A. montana*, which is similar to the direction we hypothesize for *L. rectus*, though with much younger dating.

An example of molecular clock estimate suggesting an ancient Western/Eastern Mediterranean differentiation is provided by Casimiro-Soriguer [43] for the monospecific herbaceous legume *Erophaca* Boiss. (Leguminosae). Casimiro-Soriguer [43] concluded that *Erophaca* is one of the many Tertiary relicts that form part of the present Mediterranean flora. The most recent common ancestor of the eastern and western Mediterranean subspecies of *Erophaca baetica* Boiss. was dated using relaxed molecular clock as 11.9 Ma, although with a large 95% confidence interval, 3.3–19.1 Ma, from the Late Miocene to the Early Pliocene. This estimate approaches our estimates of differentiation in *L. rectus* and *L. hirsutus*. Similar temporal patterns of infraspecific differentiation was revealed in the Mediterranean palm *Chamaerops humilis* L. [46]. As revealed by Hardion et al. [47], the origin of the Mediterranean thorny cushion-like xerophytes from *Astragalus* L. sect. *Tragacantha* DC. (Leguminosae) takes root in the middle of the Pliocene (4.4 Ma), between the Messinian salinity crisis and the onset of the Mediterranean climate.

Ackerly [48] suggested that the distinction between the age of traits and the age of taxa should be made while analyzing historical biogeorgaphy of Mediterranean floras. In this respect, it is puzzling that it is not easy to formulate which morphological traits are associated with diversification of *Lotus* in the Mediterranean region. For example, plants of *L. strictus* s.l. from non-Mediterranean part of the range (described as *L. strictus* Fisch. et C.A.Mey. s. str. from Armenia and *L. albus* Janka from Bulgaria), are morphologically similar to those from the Mediterranean part (described as *L. thermalis* Boiss.). At least, it is unlikely that characters once used to segregate *L. albus* (e.g., white corolla color) can be used as markers of any climatic adaptation. Apparently, there is only one species of *Lotus* that clearly fits the criteria of a Mediterranean habit, namely *L. fulgurans*. This is a dwarf thorny shrub from the Balearic islands. The climatic-based nature of its habit is further supported by the occurrence of an externally similar species of the same tribe, *Anthyllis hystrix* (Willk. ex Barceló) Cardona, Contandr. & Sierra that occurs on the same archipelago [49,50]. Our inferred divergence time estimate of *L. fuglugans* and *L. germanicus* is well below 2.8 Ma. Thus the estimated age of *L. fulgurans* agrees with the idea that its characteristic habit evolved as an adaptation to the current Mediterranean type of climate.

### 3.2. Evolutionary Histories of Individual Species Reveal Common Biogeographic Patterns

***Lotus strictus*** is a tap-rooted perennial herb growing in saline, marshy plains, drying up in summer, wet saline meadows and sands, lake shores, near thermal springs, at altitudes from 300 to 1300 m [51,52,53]. The species has a scattered distribution. It occurs in Inner Anatolia (provinces Konya, Kayseri, Erzincan, Ağri, Denizli), Bulgaria, Armenia (along the Araxes River), Eastern Kazakhstan (Pavlodar prov., the Urdzhar River, Lake Alakol), Altai krai of Russia, and Dzungaria (Northwest China, Northern Xinjiang) [17,51,54,55]. It is obviously absent from Greece. According to the Flora of Greece Web (http://portal.cybertaxonomy.org/flora-greece/), this species was reported for NE Greece (“Thra” in Hayek [56], p. 882, sub *Lotus strictus*), but no material has been seen by the author, and the records probably refer to glabrous forms of *L. hirsutus* (see Reference [57], p. 109).

Our sampling of *L. strictus* covers Anatolian, Bulgarian and Altai-Kazakhstan parts of its distribution range. Transcaucasian and Dzungarian parts of the range were not sampled. The Transcaucasian (Armenian) localities lie between the Anatolian and Altai-Kazakhstan parts, so we may suppose that they presumably have plastid haplotypes intermediate between the two groups of haplotypes revealed in Anatolian-Bulgarian and Altai-Kazakhstan groups. This hypothesis needs testing using plant material form the Transcaucasian part of *L. strictus* range. The Dzungarian part of the distribution range is located very close to the Altai-Kazakhstan part, so we may suppose that Dzungarian plants of *L. strictus* may possess plastid haplotypes of the same group. This assumption also needs verification using additional sampling from Northern Xinjiang.

The results obtained in the present study suggest that the distribution range of *L. strictus* was large and then experienced reduction and fragmentation. These processes led to population size decline and extinction of the haplotypes intermediate between two haplogroups. Our data allowed to suppose that the ancestral haplotypes of *L. strictus* putatively originated from the Anatolian part of the range and then the species spread to Transcaucasian and Altai-Kazakhstan-Dzungarian parts.

The extant range of *L. strictus* is intriguing. There is an impressive gap of almost 3000 km between the localities in Armenia (that closely approach those in eastern Turkey [58]) and closest localities of the species in the Eastern part of Kazakhstan (to the East of Irtysh valley in the North and Lake Alakol in the south [59]). It is unlikely that appropriate habitats are lacking within this gap, as well as in NW Kazahkstan and SW European Russia, where saline lakes are rather common. Based on our dated phylogeny, one may speculate that dispersal of *L. strictus* to the East took place in Late Miocene along the southern shore of Eastern Paratethys, a marine basin that still existed by that time (e.g., Reference [60]) and apparently prevented a direct migration northwards. The extant range of *L. strictus* may be related to margins of the Eastern Paratethys.

***Lotus rectus*** is a perennial that grows along the margins of water courses, damp and bushy places, in preferably basic substrates at altitudes from 0 to 1300 m [61,62]. The species has a wide Mediterranean distribution. It occurs in Portugal and in all countries around the Mediterranean Sea, except for Egypt and the countries of the former Yugoslavia (Slovenia, Croatia, Montenegro) [34]. Our sampling rather well covers the European part of *L. rectus* distribution range (except for Portugal, Sardinia, Sicily, and Greece) and its Asian part. The African part of the range is insufficiently represented in our study and includes a sample from Morocco. Our results imply the east-west phylogeographic differentiation of *L. rectus*, which presumably started at 4.94 Ma, soon after the Messinian crisis. The nature of genetic variability suggests that *L. rectus* has repeatedly experienced fluctuations in abundance. The present phylogeographic pattern may be a result of the range reduction, which led to decrease of gene flow between its western and eastern parts. The western and eastern populations are apparently not completely genetically isolated, as evidenced by the Moroccan and two island samples, which demonstrate contrasting genetic relatedness by nrDNA ITS and plastid data. Such a longitudinal pattern corresponds to phylogeographical or even geographical break described for many Mediterranean plant species (e.g., see review in Reference [29]). The present analysis of plastid dataset of *L. rectus* allows to suppose the location of the ancestral area of this species in the Western part of its range (i.e., in the Iberian peninsula) and its further distribution from the Western to Eastern Mediterranean.

***Lotus hirsutus*** is a sub-shrub or small shrub growing on calcareous slopes and cliffs, dry hills, macchie, pine forests at altitudes from 0 to 900 m. This species, like *L. rectus*, has an extensive Mediterranean range. It occurs in Portugal and in all countries around the Mediterranean Sea, except for Egypt, Tunisia and Morocco [34]. Our sampling covers well the European part of its distribution range, but only samples from Anatolia were available for the Asian part. Samples from Africa (Libya and Algeria) and large islands (Corse and Sardinia) were not available. According to our dated phylogeny, based on ITS, separation between *L. dorycnium* complex and *L. hirsutus* may happen at about 5.55 Ma and the divergence of *L. hirsutus* into western and eastern geographical groups could occur about 4.16 Ma. Genetic variation pattern of *L. hirsutus* plastid dataset implies that diversification of the species likely started in the eastern part of the range, in Anatolia, from where the species spread to the western part (the Iberian peninsula), where it also experienced a later divergence. The third plastid evolutionary branch of *L. hirsutus* is scattered within the eastern part of the range (from Turkey to France) and demonstrates incomplete lineage sorting with *L. dorycnium* complex. It can be assumed that *L. hirsutus*, like *L. rectus*, exhibits longitudinal phylogeographic differentiation, but with the opposite direction of distribution from east to west.

***Lotus graecus*** is a perennial herb with root suckers [17], growing on roadsides, open slopes, macchie, coniferous and deciduous forests at altitudes from 0 to 2000 m [52,61,63]. It is distributed in Greece, Bulgaria, Turkey, the Caucasus, and the Crimea. The main part of the range is located around the Black Sea reaching the East Mediterranean in the South. Our samples of *L. graecus* sufficiently well cover its entire range. The phylogeographic analysis of the *L. graecus* plastid dataset suggested a relatively young age of the species, which has recently undergone demographic expansion, as evidenced by the presence of one widely distributed central haplotype and several derived haplotypes. Both ITS and plastid data clearly indicate that two Turkish endemics, *L. axilliflorus* and *L. sanguineus*, are related to *L. graecus*. *Lotus axilliflorus* is a perennial occuring in oak scrub on marly soil in SW Anatolia (Burdur prov.) [61]. *Lotus sanguineus* is a perennial herb with a very restricted distribution in Southern part of Central Anatolia, near Bucakkışla in Karaman. It grows on south-facing slopes on calcareous substrate, in open *Pinus brutia* forests and maquis, at altitudes from 350 to 900 m [64]. According to the results on ITS dating phylogeny obtained in the present study, the separation of *L. sanguineus* from *L. graecus* and *L. axilliflorus* can be estimated at about 6.38 (2.715, 9.641) Ma, before the Messinian crisis, and two latter species diverged from each other much later, at about 2.81 (0.776, 5.042) Ma.

## 4. Materials and Methods

### 4.1. Plant Material

The plant material used for the present study was sampled from herbarium specimens stored in several large Herbaria (ALTB, ANK, GAZI, LE, MA, MHA, MW, NS, P, SO, SOM, WAG, ZA). In total, we analyzed 104 specimens, which belong to 32 species and thirteen sections of the genus *Lotus* with expanded representation of species of *Bonjeanea* group (i.e., *L. hirsutus*, *L. rectus*, *L. strictus*, and *L. graecus*). *Cytisopsis pseudocytisus* (Boiss.) Fertig, *Hammatolobium kremerianum* (Coss.) Müll. Berol., and *Tripodion tetraphyllum* (L.) Fourr. were used as an outgroup. For 70 specimens, the sequences of all the studied DNA regions or some regions were obtained in this study, the rest of the sequences were taken from GenBank. Voucher information and GenBank accession numbers are presented in Appendix A. Distribution maps of the studied specimens were constructed using SimpleMappr [65].

### 4.2. DNA Extraction, Amplification and Sequencing

DNA was extracted from dry leaves taken from herbarium (ca. 20 mg of leaf tissue) with NucleoSpin Plant II kit (Macherey-Nagel, Germany) according to the manufacturer’s instructions or using the CTAB (cetyl trimethylammonium bromide) method [66].

The sequences of the nrITS were amplified with primers NNC-18S10 и С26А [67] and universal primers ITS2 and ITS3 [68]. The sequences of *trn*L-*trn*F intergenic spacer (IGS) and *trn*L intron of plastid DNA were amplified using standard primers ‘c’, ‘d’, ‘e’ and ‘f’ [69]. The sequences of *rps*16 intron of plastid DNA were amplified using primers rpsF and rpsR2 [70]. We also used forward internal primer rps16internF [71] in a combination with rpsR2 for amplification of the second (3′) part of *rps*16 intron. However, the internal reverse primer rps16internR [71] in a combination with rpsF gave no amplification in *Lotus* samples. To amplify the first (5′) part of *rps*16 intron, we designed a pair of primers specific to *Lotus*, Lot-rps16-F (5′-GTGGTAAAAAGCAACGTGCG-3′) and Lot-rps16-intR (5′-GCTTTTCCTTGAATCATTGGGT-3′). The primers were developed using the Primer-BLAST [72] software based on the complete sequence of *Lotus japonicus* (Regel) K.Larsen plastome (GenBank accession number NC_002694.1). Newly developed primer Lot-rps16-intR in a combination with either rpsF or Lot-rps16-F produced good results in many *Lotus* species and allowed to amplify sequences about 500 bp long.

PCRs were performed in a 0.02 ml mixture containing 10–20 ng DNA, 3.2 pmol of each primer and MasDDTaqMIX (Dialat LTD, Moscow, Russia) containing 0.2 mM of each dNTP, 1.5 mM MgCl2, and 1.5 units of SmarTaqDNA polymerase. Amplification of nrITS region was performed under the following conditions: hold 94 °С, 3 min; 94 °С, 30 s; 57 °С, 40 s; 72 °С, 60 s; repeat 30 cycles; extend 72 °С, 3 min. Amplification of *trn*L intron, *trn*L-*trn*F IGS, and *rps*16 intron regions of chloroplast DNA (cpDNA) was performed under the following conditions: hold 94 °С, 3 min; 94 °С, 30 s; 58 °С, 40 s; 72 °С, 60 s; repeat 30 cycles; extend 72 °С, 3 min.

PCR products were purified using Cleanup Mini kit (Evrogen, Moscow, Russia) following the manufacturer’s instructions. Direct sequencing was performed on the ABI PRISM 3100 genetic analyzer (Applied Biosystems, Foster City, CA, USA), using ABI Prism BigDye Terminator Cycle Sequencing Ready Reaction Kit v. 3.1 for cycle sequencing reactions following the manufacturer’s instructions. Forward and reverse strands of all samples were sequenced. The polymorphism of ITS within one specimen was detected by direct sequencing (without cloning), by the presence of double peaks on electropherogram.

The sequences were aligned using MAFFT version 7.215 [73,74] and then adjusted manually in BioEdit version. 7.2.5 [75]. The matrices of *rps*16 intron and *trn*L-F cpDNA regions were combined into a single matrix. Gap-rich and ambiguous positions were excluded from the analyses. The aligned data matrices are presented in on-line Supplement (Datasets S1–S2).

### 4.3. Phylogenetic Analyses

The Maximum Likelihood analyses were performed with MEGA X [76] with GTR+Г+I model of nucleotide substitutions (the general time–reversible model with the presence of invariable sites and substitution rate heterogeneity) for the plastid dataset sequences and GTR+Г model for nrITS sequences. The models were determined as the best choice for corresponding datasets following the Model Selection option implemented in MEGA X based on the corrected Akaike information criterion (AICc). Nonparametric bootstrap method with 500 replications was used for branch support assessment.

The Bayesian inference was performed using MrBayes v. 3.2.6 [77] considering the optimal model of nucleotide substitutions selected by AICc in PAUP version 4.0a [78] for each marker: SYM+Г (symmetrical model with substitution rate heterogeneity) for nrITS, and GTR+Г for plastid data. The Bayesian analysis used two independent runs of 20 million generations and four chains sampling every 1000th generation. Non-convergence assessment and burn-in estimation was carried out in VMCMC ver. 1.0.1 [79]. The first two million generations were discarded as burn-in and the remaining trees from both runs were combined in a 50% majority-rule consensus tree.

Phylogenetic relationships among the cpDNA haplotypes were reconstructed using statistical parsimony analysis as implemented in TCS v1.2 [80]. Long indels were reduced to one character, then gaps were treated as fifth state. TCS networks of haplotypes were constructed separately for *L. rectus*, *L. strictus*, *L. hirsutus*, and *L. graecus*. *L. corniculatus* and *L. dorycnium* were used as outgroups. Haplotype networks were then visualized using the online program tcsBU [81]. Parameters of genetic variability were calculated using DnaSP 6 software [82].

### 4.4. Dating Analyses

For dating the *Lotus* phylogeny, we used partially reduced dataset of *Lotus* nrITS sequences (45 accessions representing 30 species) but with an enlarged outgroup, including 13 genera of the tribe Loteae, two species of *Sesbania*, three species of *Robinia* and *Coursetia glandulosa* A.Gray from Robineae (nrITS sequences of the outgroup were retrieved from GenBank; accession numbers are presented in Appendix B). The aligned data matrix is presented in on-line Supplement (Supplementary Materials, Dataset S3). For divergence time estimation, the nrITS dataset was analyzed using BEAST version v.2.6.3 [83]. The best performing substitution model (SYM + Г) was implemented along with the Yule [84] and Birth–Death [85] speciation priors. Two clock models (strict clock and uncorrelated relaxed clock with a log-normal distribution) were tested using the nested sampling approach [86] implemented in the NS package of BEAST 2, and the strict clock model was rejected basing on marginal likelihood estimations (Table 3). We used two calibration points in phylogenetic dating: a normal distribution (mean = 48.3, SD = 1) was applied to the root age according to the Robinioid crown age estimated by Lavin et al. [20], and a log-normal distribution (mean = 5, S = 0.7) with a minimum age constraint (offset) at 34 Ma to the *Robinia* stem age according to *Robinia* L. wood fossil (discussed in [20,26]). Two Markov chains Monte Carlo were run for 40 million generations, trees and parameters were sampled every 4000 generations. Tracer ver. 1.7.1 [87] was used to assess the chain convergence, burn-in and the effective sample sizes. The effective sample size exceeded 200 in all analyses, and burn-in was set to 10%. Before conducting the dating analyses, sampling from prior distributions only was performed to be sure that the marginal distribution of the priors reflected our intended settings.

## 5. Conclusions

The results of the present phylogenetic study of *Lotus* section *Bonjeanea*, like previously obtained data (Degtjareva et al., 2006, 2008; Kramina et al., 2016), clearly demonstrated the non-monophyletic nature of the section. Our data suggest that *L. rectus* and *L. strictus* differentiated at the early stages of evolution of the genus *Lotus* and represent two separate evolutionary lineages, which can probably be considered as two distinct sections. Our preliminary morphological analysis of herbarium specimens did not reveal clear morphological differences between western and eastern populations of *L. rectus*, as well as between Anatolian-Bulgarian and Altai-Kazakhstan populations of *L. strictus*, despite significant genetic differences. Currently, the differentiation observed within each of these two species is probably consistent with the concept of cryptic species; however, both species require more careful morphological analysis. The present study further supports our earlier—quite unexpected—findings (Kramina et al., 2016) on close relationships between *L. hirsutus* and members of the *L. dorycnium* complex with strong incongruence between plastid and nuclear data.

## Figures and Tables

**Table 1:** Morphological characteristics of *Lotus rectus, L. hirsutus*, and *L. structus* compared to those of species traditionally placed in *Lotus* and *Dorycnium*.

Taxa	*Lotus dorycnium*, *L. herbaceus*, *L. germanicus*, *L. fulgurans*, *L. graecus*, *L. axilliflorus*, *L. sanguineus* (Species Formerly Considered as Typical Members of *Dorycnium*)	*L. rectus*	*L. hirsutus*	*L. strictus*	Other Species of *Lotus*	Outgroups
Taxonomic placement in Sokoloff (2003b) and Degtjareva et al. (2006)	*Lotus* section *Dorycnium*	*Lotus* section *Bonjeanea*			*Lotus* sections *Benedictella*, *Chamaelotus*, *Erythrolotus*, *Heinekenia*, *Krokeria*, *Lotea*, *Lotus*, *Ononidium, Pedrosia*, *Rhyncholotus*, *Tetragonolobus*	*Tripodion, Hammatolobium*, *Cytisopsis*
Leaf rachis	Almost always absent	Present	Present or absent	Present or absent	Usually present, but absent in some species of the Southern Clade	Present or absent
Position of sterile bract (see Sokoloff et al., 2007)	Often separated from partial inflorescence by a stalk	Often separated from partial inflorescence by a stalk	Often separated from partial inflorescence by a stalk	At the base of partial inflorescence	At the base of partial inflorescence	At the base of partial inflorescence (*Hammatolobium*, *Tripodion*) or absent (*Cytisopsis*)
Flowers and partial inflorescences	Partial inflorescences more commonly many-flowered (6–30 flowers often arranged on inflorescence axis in more than one whorl)		Partial inflorescences commonly few-flowered (a whorl of 1–8 flowers).			
Flower size	<7 mm	<7 mm	>7 mm	>7 mm	> or <7 mm	>7 mm
Petal colour	Never yellow	Never yellow	Never yellow	Yellow to white	Yellow or not yellow	Yellow or not yellow
Keel shape	Obtuse	Obtuse	Rostrate	Rotrate	Rostrate	Rostrate or obtuse
Distal parts of wing petals	Adhering together	Free	Free	Free	Free or (in some members of the Southern Clade) adhering together	Free
Outgrowths in distal parts of wing petals	Present	Absent	Absent	Absent	Absent	Absent
Style surface	Smooth	Smooth	Smooth	Smooth	Papillose	Smooth
Fruits	1-seeded (few-seeded in *L. graecus*)	Many-seeded	Many-seeded	Many-seeded	Usually many-seeded	Two- to many-seeded
Brown cells in endocarp	Usually present	Present	Present	Present	Absent	Present or absent

**Table 2:** Estimated divergence times of the major clades within Loteae and Lotus, obtained with an uncorrelated lognormal clock model under a Birth-death speciation process.

Node	nrITS Node Defined as MRCA or Crown Clade of	Mean Age (Ma)	CI Values (95% HPD)
A	Loteae	29.11	18.286–34.82
B	*Lotus* + *Cytisopsis* + *Tripodion*	20.71	12.907–25.643
C	*Lotus*	15.87	9.995–19.813
D	*Lotus* Northern clade 1	12.47	7.526–15.784
E	*Lotus* Northern clade 2	12.4	7.228–16.036
F	*Lotus* Southern clade	10.21	5.827–13.356
G	*Lotus* section *Dorycnium* (incl. *L. hirsutus*)	9.83	5.447–13.151
H	*L. sanguineus* + *L. graecus* + *L. axilliflorus*	6.38	2.715–9.641
I	*L. graecus* + *L. axilliflorus*	2.81	0.776–5.042
J	*L. hirsutus* + *L. dorycnium*	5.55	2.916–8.143
K	*L. fulgugans* + *L. germanicus*	1.61	0.489–2.831
L	*L. hirsutus*	4.16	1.731–6.412
M	*L. rectus* + *L. strictus*	10.58	6.124–13.964
N	*L. rectus*	4.94	2.078–7.603
O	*L. strictus*	6.1	2.815–8.768

**Table 3:** Marginal likelihoods estimated using the nested sampling approach under relaxed and strict clock models in combination with Birth-Death (BD) and Yule speciation priors.

	BD	Yule
relaxed clock	−7391.169 ± 6.251	−7386.553 ± 6.170
strict clock	−7415.723 ± 8.205	−7422.717 ± 8.211

## Supplementary Materials

The following are available online at https://www.mdpi.com/2223-7747/10/2/260/s1, Figure S1: Distribution of pairwise differences between studied sequences of plastid DNA of the species: A–*L. rectus*; B–*L. strictus*; C–*L. hirsutus*; D–*L. graecus*. Dataset S1: nrITS dataset. Dataset S2: combined plastid dataset (*rps*16 and *trn*L-F). Dataset S3: nrITS dataset used for Loteae divergence dating analyses.

## Appendix A

Taxa, sample code, locality, voucher information (herbarium code) for the material included in the phylogenetic analyses. GenBank accession numbers are given for the three markers sequenced, *rps*16 intron, *trn*L-F, ITS (new sequences indicated by an asterisk); Herbarium codes according to Index Herbariorum. An n-dash denotes a missing marker (GenBank accession numbers of new sequences will be inserted in the final version of the article).

***Cytisopsis pseudocytisus* (Boiss.) Fertig; 7**; Turkey, C1, Muğla, Datça, Knidos, 29–31.V.1995, *A.P.Khokhryakov & M.T.Mazurenko s.n.* (MHA); HM468299; MK751647; AY325282; ***Hammatolobium kremerianum* (Coss.) C.Muell.; 643**; Morocco, *Podlech 51378* (MHA); KT262933; MK751648; KT250926; ***Lotus aegaeus* Boiss.; 427**; Turkey, C3, Antalya Korkuteli, Termesos, Buyukkumluca, Cakıllı gecidi, 04.VI.1995, *A.P.Khokhryakov & M.T.Mazurenko 1135* (MHA); KT262865; MK751649; DQ160276; ***Lotus angustissimus* L.; 472**; Australia, Norfolk Island, introduced, 14.X.1999, *B.M.Waterhouse 5510* (NSW); KT262868; MF158217; DQ166243; ***L. axilliflorus* (Hub.-Mor.) D.D.Sokoloff; 4796**; Turkey, C2 Burdur, Yeşilova, Salda gölü, 23.V.1993, *H.Duman, F. Karavelioğulları 4796*, (GAZI); MW498318*; MW470872*; MW412841*; ***L. axilliflorus* (Hub.-Mor.) D.D.Sokoloff; 5089**; Turkey, C2 Burdur, Yeşilova, Salda gölü, 12.VIII.1993, *H.Duman, Z.Aytac & Dцnmez 5089* (GAZI); MW498319*; MW470873*; MW412842*; ***L. axilliflorus* (Hub.-Mor.) D.D.Sokoloff; 941**; Turkey, *Duman et al. 5089* (E); KT262869; MN553691; KT250852; ***Lotus broussonetii* Choisy ex Ser.; 21**; Cultivated at Royal Botanic Gardens, Kew: introduced from Canary Is.; KT262872; MK751653; DQ160278; ***Lotus conimbricensis* Brot.; 485**; Spain, Badajoz, Almendral, 27.IV.1966, *Segura Zubizarreta 960* (Z); KT262874; MF158231; FJ411114; ***Lotus corniculatus* L.; L7**; Russia, Moscow prov., Lutzino, 03.VII.2008, *Kramina 74-7* (MW); KT262876; MW470874*; JF784200 & JF784201; ***Lotus discolor* E.Mey.; 444**; Cameroon, *S.Lisowski B-3330* (BR); KT262880; MK751659; DQ160288; ***Lotus dorycnium* L.; D3**; Spain, Valencia, Algar, 18.IV.1995, *J.Riera, J.Güemes & E.Estrelles 17073* (H); KT262884; MK751662; KT250862; ***Lotus herbaceus* (Vill.) Jauzein ssp. *gracilis* (Jord.) Jauzein; D8**; France, dép. Pyrénées-Orientales, Canet, 02.VII.1981, *J.Lambinon, R.Renard & L.Smeets 81/287* (H); KT262881; MK751682; KT250859; ***Lotus dorycnium* L. (*Dorycnium pentaphyllum* ssp. *suffruticosum*); D7**; France, Alpes-Maritimes, Blausasc, 14.V.1977, *A. Charpin & P. Hainard 9350* (H); KT262883; MK751660; KT250861; ***Lotus dorycnium* L. (*Dorycnium pentaphyllum* ssp. *transmontanum*); D2**; Portugal, prov. Trás-os-Montos, Mogadouro, 25.V.1988, *R. Auriault 14166* (H); KT262882; MK751661; KT250860; ***Lotus edulis* L.; 623**; Cyprus, 10 km to W from Limassol, 13.III.2004, *Seregin & Sokoloff A-280* (MW); KT262885; MK751663; KT250863; ***Lotus eriophthalmus* Webb & Berthel.; ERIO**; Spain, Tenerife, Cultivated at Botany Dept. of University of La Laguna, 11.V.1984, *A.Gharpin M. del Asco 185745* (MA 318437); MW498320*; MW470875*; MW412843*; ***Lotus eriosolen* (Maire) Mader et Podlech; 414**; Morocco, prov. Ourzazate, 06.IV.1995, *D. Podlech 52619* (M); KT262886; MK751664; DQ160281; ***Lotus fulgurans* (Porta) D. D. Sokoloff; 937**; United Kingdom, Cultivated at Royal Botanic Gardens, Kew, 2010: origin Spain, Balearic Is.; KT262887; MF314954; KT250865; ***Lotus germanicus* (Gremli) Peruzzi; D1**; Slovenia, Polhograjsko Hribovje, prope Govejek, supra vicum Medvode, 19.VI.1973, *D.Trpin & T.Wraber 9852/3* (H); KT262889; MK751666; KT250868; ***Lotus germanicus* (Gremli) Peruzzi; D4**; Germany, Bayern, Oberbayerische Hochebene, n. München, 06.VII.1991, *H. Kalheber 91-0625* (H); KT262890; MK751667; KT250869; ***Lotus germanicus* (Gremli) Peruzzi; D5**; Montenegro, 40 km NNE of Nikšic, Žabljak, *P.Uotila 10652* (H); KT262891; MK751668; KT250870; ***Lotus glinoides* Del.; 461**; Egypt, 7.V.1962, *Bochantsev s.n.* (LE); KT262892; MK751677; DQ160282; ***Lotus graecus* L.; 2459**; Turkey, B3 Kütahya, Dumlupınar, Gökdağ, Akdene mevkii, 22.VII.1983, *M.Vural, F.Maluen 2459* (GAZI); MW498321*; MW470876*; MW412844*; ***Lotus graecus* L.; 2504**; Turkey, A4 Ankara, Kızılcahamam, c. 65 km N of Ankara, 27.VII.1989, *R.M.Nesbitt & D.J.Samuel 2504* (GAZI); MW498322*; MW470877*; MW412845*; ***Lotus graecus* L.; Ab1**; Abkhazia, lake Ritsa, 07.VI.2019, *M.V.Lysova, S.V.Polevova Ab1* (MW); MW498323*; MW470878*; MW412846*; ***Lotus graecus* L.; Ab3**; Abkhazia, Sukhumi highway, roadside, 07.VI.2019, *M.V.Lysova, S.V.Polevova Ab3* (MW); MW498324*; MW470879*; MW412847*; ***Lotus graecus* L.; Ca1, Ca2**; Crimea, Vinogradnoye, mount Castell, *T.E.Kramina Ca1-2* (MW); MW498325*-MW498326*; MN553692–MN553693; MN545698–MN545699; ***Lotus graecus* L.; D10**; Greece, East Macedonia, Thasos, Glifada, 18.V.1986, *T.Raithalme s.n.* (H); KT262894; MK751678; KT250877; ***Lotus graecus* L.; D9**; Turkey, A3, Bolu, Düzce-Akçakoca, 24.V.1990, *R*.*Lampinen 7871* (H); KT262893; MK751679; KT250876; ***Lotus graecus* L.; GL1**; Russia, Krasnodarsky krai, Dzhanhot, 04.VI.2016, *A.O.Viricheva s.n.* (MW); MW498327*; MN553694; MN545701; ***Lotus graecus* L.; GR1**; Turkey, A8 Trabzon, Sürmene, 20.VI.1984, *Vural 2938* (GAZI); MW498328*; MW470880*; MW412848*; ***Lotus graecus* L.; Ist1**; Turkey, A2 İstanbul, Tuzla, campus of the Sabancı University, 07.V.2017, *A.A.Sinyushin s.n.* (MW); MW498329*; MW470881*; MW412849*; ***Lotus graecus* L.; So3**; Russia, Krasodarsky krai, Sochi, between Volkonskaya and Soloniki, 05.VI.2017, *Kuturova M.V. So3* (MW); MW498330*; MN535054; MN545704; ***Lotus graecus* L.; So6**; Russia, Krasodarsky krai, Agura River, Orlinye skaly, 03.VI.2017, *M.V.Kuturova So6* (MW); MW498331*; MW470882*; MN545706; ***Lotus graecus* L.; Tr1**; Turkey, A2 İstanbul, 2 km W of İstanbul Airport, 26.05.2019, *Lysova & Kramina Tr1* (MW); MW498332*; MW470883*; MW412850*; ***Lotus graecus* L.; Tr2**; Turkey, A2 İstanbul, 4.5 km N of Karacaköy, 26.05.2019, *Lysova & Kramina Tr2* (MW); MW498333*; MW470884*; MW412851*; ***Lotus graecus* L.; UT4, UT7**; Russia, Krasnodarsky krai, Anapsky distr., lake Sukhoy liman, 29.V.2016, *M.V.Kuturova UT4, UT7* (MW); MW498334*, MW498335*; MW470885*, MW470886*; MN545710, MN545712; ***Lotus halophilus* Boiss. & Spruner; 431**; Greece, Karpathos, Pigadia, 19-Apr-1984, *Th.Raus 9307* (MHA); KT262896; MK751680; KT250879; ***Lotus herbaceus* (Vill.) Jauzein; D6**; Austria, Steirisches Hügelland, Steiermark, Umgebung von Radkersburg, 7.VII.1976, *H.Mayrhofer & H.Teppner s.n.* (H); KT262898; MK751681; KT250882; ***Lotus hirsutus* L.; 3052322**; Greece, Macedonia, Kalithea, 00.IV.1995, *G. Van Buggenhout 17072* (P 03052322); MW498337*; MW470888*; MW412853*; ***Lotus hirsutus* L.; 3052323**; Spain, Prov. Teruel, Mosqueruella, 24.V.1992, *C.Fabregat & S.Lуpez s.n.* (P 03052323); MW498338*; MW470889*; MW412854*; ***Lotus hirsutus* L.; 3052351**; France, Aude, Massif de la Clape, 16.V.1975, *B. de Retz 71072* (P 03052351); MW498340*; MW470891*; MW412855*; ***Lotus hirsutus* L.; 1**; Spain, prov. Teruel, Mosqueruella, 24.V.1992, *C.Fabregat & S.L**у**pez s.n.* (MHA); MW498341*; MW470892*; MW412856*; ***Lotus hirsutus* L.; 1841**; Turkey, C2 Muğla, Marmaris, Bağli Tepe Livari, 27.VI.1997, *H.Sağban 1841* (GAZI); MW498342*; MW470893*; MW412857*; ***Lotus hirsutus* L.; 2058**; Turkey, C5 Adana Karataş, Yumurtalık Lagünü, 19.IV.1998, *H.Sağban 2058* (GAZI); MW498343*; MW470894*; MW412858*; ***Lotus hirsutus* L.; 3052321**; Spain, prov. Huesca, Rodellar, Sierra de Rufas, 22.V.1970, *P.Montserrat JACA 108370* (P 03052321); MW498336*; MW470887*; MW412852*; ***Lotus hirsutus* L.; 3052324**; Spain, Prov. Alicante, commune de Javea, 18.IV.1979, *P.Villaret 19466* (P 03052324); MW498339*; MW470890*; —; ***Lotus hirsutus* L.; 343816**; Spain, Gerona, Girones, Canet d’Adri, 16.V.1986, *E.Castells, J.Pedrol s.n.* (MA 343816-2); MW498344*; MW470895*; MW412859*; ***Lotus hirsutus* L.; 4**; Montenegro, 30 km of Titograd, NE of Petrovac, 19.VI.1971, *P.Uotila 10633* (MHA); MW498345*; MW470896*; MW412860* & MW412861*; ***Lotus hirsutus* L.; 609**; Spain, near Barcelona, July 2006, *Beer & Beer s.n.* (MW); KT262902; MK751683; KT250886; ***Lotus hirsutus* L.; 626236**; Spain, prov. Huesca, Rodellar, Sierra de Rufas, 22.V.1970, *P.Montserrat s.n.* (MA 626236); MW498346*; MW470897*; MW412862*; ***Lotus hirsutus* L.; 7**; Turkey, C1 Aydın, Menderes Nehri, Bafa Gölü, 28.V.1995, *A.P.Khokhryakov, M.T.Mazurenko s.n.* (MHA); MW498347*; MW470898*; MW412863*; ***Lotus hirsutus* L.; D11**; Turkey, A1 Çanakkale, Yalova-Eceabat, 15.V.1990, *R.Lampinen 7355* (H); KT262899; MN553705; KT250883; ***Lotus hirsutus* L.; D12**; Greece, East Macedonia, Thasos, Glifada, 19.V.1986, *T.Raithalme s.n.* (H); KT262901; MK751684; KT250885; ***Lotus hirsutus* L.; D13**; Croatia, Korcula island, SW of Pupnat, 23.VI.1971, *L.Hämet-Ahti 2225* (H); KT262900; MK751685; KT250884; ***Lotus hirsutus* L.; GC3**; Greece, Kerkyra, Benitses, 25.VIII.2018, *D.D.Sokoloff s.n.* (MW); MW498348*; MW470899*; MW412864*; ***Lotus hirsutus* L.; HIRS1**; Spain, Madrid, Alcala de Henares, 07.VII.1996, *E.Soberino Vesperinas s.n.* (MA 582050); MW498349*; MN553706; MN545736; ***Lotus hirsutus* L.; HIRS2**; Italia, Sicilia, Ragusa, 09.VI.2000, *Alvares et al. IA 1784* (MA 645120); MW498350*; MN553707; MN545737; ***Lotus hirsutus* L.; HIRS3**; Croatia, Lokrum, 15.V.1977, *S.Heéimovié s.n.* (ZA); MW498351*; MW470900*; MW412865*; ***Lotus hirsutus* L.; HIRS4**; Croatia, island Biševo, 25.VII.1981, *B.Korica s.n.* (ZA); MW498352*; MW470901*; MW412866*; ***Lotus hirsutus* L.; HIRS5**; Croatia, Zakovae (Šibenik), 29.V.1997, *M.Milović s.n.* (ZA); MW498353*; MW470902*; MW412867*; ***Lotus hirsutus* L.; HIRS6**; Italy, Portofino Vetta, 26.V.1961, *P.Jovet s.n.* (P 00963521); MW498354*; MW470903*; MW412868*; ***Lotus hirsutus* L.; HIRS8**; Italy, Napoli, Capri, 5.VI.1975, *E.Meijer Drees It 713* (WAG 1017361); MW498355*; MW470904*; MW412869*; ***Lotus hirsutus* L.; Sp4**; Portugal, Algarve, Vila do Bispo, 06.VI.2001, *L.Medina, S.Nisa, M.Pardo de Santayana s.n.* (MA); MW498356*; MW470905*; MW412870*; ***Lotus laricus* Rech.f., Aellen & Esfand.; 455**; Abu Dhabi, Abu Dhabi Island, Al Mushrif Palaca, 04.V.1982, *R.A.Western 275* (E); KT262906; MK751687; DQ166233; ***Lotus maculatus* Breitf.; 958**; Canary Is. (cult.), Tenerif. Municipio de la Orotava, Puerto de la Cruz, 14.IV.2000, *H.Väre 10894* & *H.Kaipiainen* (H 1702795); KT262907; MK751688; KT250890; ***Lotus ononopsis* Balf. f.; 453**; Yemen, Muqadrihon Pass, c. 10 km SW of Hadiboh, 26.I.1990, *A.G.Miller et al. 10097* (E); KT262909; MK751690; DQ166219; ***Lotus parviflorus* Desf.; 469**; Spain, Talavera-de-la-Reina, 09.V.1987, *Segura Zubizarreta 34.567* (MHA); MW498357*; MF314955; DQ166230; ***Lotus pedunculatus* Cav.; 437**; Spain, Soria, Santa Inйs, 18.VII.1972, *Segura Zubizarreta s.n.* (LE); KT262910; MF158224; DQ166222; ***Lotus rectus* L.; 3027003**; Marocco, Prov. de Beni-Mellal, Cascades d’Ouzoud, 06.VII.1989, *Podlech 47695* (P 03027003); MW498358*; MW470906*; MW412871*; ***Lotus rectus* L.; 2**; Israel, Philistaean Plain, env. of Palmahim swamps, 22.VI.1958, *M.Zohary & I.Amdursky 639* (MHA); MW498359*; MW470907*; MW412872*; ***Lotus rectus* L.; 3**; France, Villeneuve-les-Maguelonne, 11.VII.1975, *A.Dubius 7516* (MHA); MW498360*; MW470908*; MW412873*; ***Lotus rectus* L.; 401**; Lebanon, on the bank of the Nahr el Kalb, 05.VI.1959, *T.D.Maitland 401* (LE); MW498361*; MW470909*; MW412874*; ***Lotus rectus* L.; 5**; Spain, Huelva, Hinojos, 28.VI.1975, *B.Cabezufo & S.Silbestre 2695/75* (MHA); MW498362*; MW470910*; MW412875*; ***Lotus rectus* L.; 9300**; Turkey, C2 Muğla, Köyceğiz, Beyobası Köyü, 24.V.1991, *A.Güner, M.Vural, H.Sağban 9300* (GAZI); MW498363*; MW470911*; MW412876*; ***Lotus rectus* L.; 955**; Crete, Retimno, VIII.2012, *Sokoloff s.n.* (MW); KT262915; MW470912*; KT250902; ***Lotus rectus* L.; REC1**; Spain, Alicante, Rio Guadalest, 02.VII.1958, *A.Rigual s.n.* (MA 373077); MW498364*; MK751693; MK780164; ***Lotus rectus* L.; REC2**; Spain, Castelló, Burriana, 04.V.2006, *R.Roselló Gimeno s.n.* (MA 741964); MW498365*; MK751694; MK780165; ***Lotus rectus* L.; REC3**; Italy, Liguria, Imperia, Vermiglia, 14.VII.2008, *Wieringa and A.Kool 6621* (WAG 8003059); MW498366*; MW470913*; MW412877*; ***Lotus rectus* L.; REC4**; France, Corse, Haute-Corse, Corsica, St.-Florent, 03.VII.1970, *J.Rammeloo 1620* (WAG 1005625); MW498367*; MW470914*; MW412878*; ***Lotus rectus* L.; REC5**; France, Languedoc-Roussellon, 22.VI.1995, *Biology Students Wageningen 95134* (WAG 1017736); MW498368*; MW470915*; MW412879*; ***Lotus sanguineus* (Vural) D.D.Sokoloff; 7521**; Turkey, C4 Karaman, Bucakkışla, 21.VI.1996, *M.Vural, N.Adıgüzel 7521* (GAZI); MW498369*; MW470916*; MW412880*; ***Lotus sanguineus* (Vural) D.D.Sokoloff; 940**; Turkey, C4 Konya, 1981, *M.Vural 1976* (E); KT262916; MN553710; KT250904; ***Lotus spectabilis* Choisy ex Ser.; SPEC**; Spain, Tenerife, Güimar, VIII.1977, *A.Santos-Ricardo 5124* (MA 839030); MW498370*; MW470917*; MW412881*; ***Lotus strictus* Fisch. & C.A.Mey.; 3845**; Turkey, B4, Tuz gölü, Aksaray-Eşmekaya sazlığı, 13.VII.1997, *M.Aydoğdu 3845* (ANK); MW498371*; MW470918*; MW412882*; ***Lotus strictus* Fisch. & C.A.Mey.; 413**; Russia, Altai Krai, Mikhaylovsky distr. 18.IX.2003, *Korolyuk s.n.* (MW); KT262923; MF158210; DQ160286; ***Lotus strictus* Fisch. & C.A.Mey.; 7069**; Turkey, B4 Aksaray, Sultanhanı, Esmekaya, 22.IX.1993, *M.Vural, H.Duman, N.Adıgüzel, F.Karavelioğulları 7069* (GAZI); MW498372*; MW470919*; MW412883*; ***Lotus strictus* Fisch. & C.A.Mey.; 7731**; Turkey, C4 Konya, Aslım Bataklığı, 26.IX.1996, *M.Vural, H.Duman, N.Adıgüzel 7731* (GAZI); MW498373*; MW470920*; MW412884*; ***Lotus strictus* Fisch. & C.A.Mey.; 923**; Kazakhstan, Pavlodar Prov., Kanonerka, 1956, *I.Povalyaeva s.n.* (MW); KT262924; MF158211; KT250914; ***Lotus strictus* Fisch. & C.A.Mey.; 9327**; Turkey, C4 Konya-Ereğli, Akgöl bataklığı, 07.IX.1992, *T.Ekim 9327* (GAZI); MW498374*; MW470921*; MW412885*; ***Lotus strictus* Fisch. & C.A.Mey.; Al1**; Russia, Altai Krai, Uglovsky distr., 6 km SW of Simonovo, 15.IX.1999, *I.Artemov*, *A.Korolyuk, N.Makunina s.n.* (NS); MW498375*; MW470922*; MW412886*; ***Lotus strictus* Fisch. & C.A.Mey.; Al2**; Russia, Altai Krai, Malinovoye ozero, 17.IX.2003, *Maslova, Khrustaleva s.n.* (ALTB); MW498376*; MW470923*; MW412887*; ***Lotus strictus* Fisch. & C.A.Mey.; Al3**; Russia, Altai Krai, Uglovsky distr., Shadrukha, 31.VII.1946, *A.Strom, V.Mochalov s.n.* (ALTB); MW498377*; MW470924*; MW412888*; ***Lotus strictus* Fisch. & C.A.Mey.; Al4**; Russia, Altai Krai, Klyuchi, 18.VII.1960, *B.Zaitzev* et al*. s.n.* (NS); MW498378*; MW470925*; MW412889*; ***Lotus strictus* Fisch. & C.A.Mey.; STR1**; Bulgaria, prope Philippopel (Plovdiv), 17.VIII.1895, *V.Střibrný s.n.* (SO 43007); MW498379*; MW470926*; MW412890*; ***Lotus strictus* Fisch. & C.A.Mey.; STR2**; Bulgaria, Plovdiv Prov., Krumovo, 14.VIII.1893, *V.Střibrný s.n.* (SO 43010); MW498380*; MW470927*; MW412891*; ***Lotus strictus* Fisch. & C.A.Mey.; STR3**; Bulgaria, Plovdiv Prov., Krumovo, 5.VIII.1896, *V.Střibrný s.n.* (SO 43011); MW498381*; MW470928*; MW412892*; ***Lotus strictus* Fisch. & C.A.Mey.; STR4**; Bulgaria, lake Burgas (lake Vaya) 1.VIII.1928, *D.Yordanov s.n.* (SO 43012); MW498382*; MW470929*; MW412893*; ***Lotus strictus* Fisch. & C.A.Mey.; STR5**; Bulgaria, Dolno Ezerovo, lake Burgas, 4.VI.1967, *V.Velchev s.n.* (SOM 154381); MW498383*; MW470930*; MW412894*; ***Lotus strictus* Fisch. & C.A.Mey.; STR6**; Bulgaria, ad Poutum, Slantchev brjag, VII.1992*, A.Petrova s.n.* (SOM 155933); MW498384*; MW470931*; MW412895*; ***Lotus strictus* Fisch. & C.A.Mey.; STR7**; Bulgaria, Yambol Prov., 2 km E of Atolovo, 30.VIII.2011, *S.Stoyanov s.n.* (SOM 170675); MW498385*; MW470932*; MW412896*; ***Lotus subbiflorus* Lag.; 470**; Italy, prov. Latina, Lazio, Pianura Pontina, 15.06.1991, *M.Iberite 15222* (MHA); KT262925; MF158212; DQ166231; ***Lotus tetragonolobus* L.; 624**; Cyprus, to E from Limassol, Amathus, 08.III.2004, *A.Seregin & al. A-110* (MW); KT262927; MK751696; HM468334; ***Tripodion tetraphyllum* (L.) Fourr.; 625**; Cyprus, 7.5 km to N from Limassol, 11.III.2004, *A. Seregin & D. Sokoloff A-240* (MW); HM468314; MK751698; HM468340.

## Appendix B

Taxa and GenBank accession numbers for nrITS sequences of the outgroup retrieved from GenBank, used in the dating analyses.

Acmispon americanus (Nutt.) Rydb., (as Lotus unifoliolatus), AF450183; Antopetitia abyssinica A.Rich., DQ166212; Coursetia glandulosa A.Gray, KT281009; Hippocrepis emerus (L.) Lassen, AF218531; Hosackia crassifolia Benth., (as Lotus crassifolius var. crassifolius) AF218523; Kebirita roudairei (Bonnet) Kramina & D.D.Sokoloff, AF450200; Ornithopus micranthus Arechav., AY325277; Ottleya oroboides (Kunth) D.D.Sokoloff (as Lotus oroboides), AF218510; Podolotus hosackioides Benth., DQ166214; Pseudolotus villosus (Blatt. & Hallb.) Ali & D.D.Sokoloff, DQ166215; Robinia pseudoacacia L., AF218538; Robinia hispida L., AF537360; Robinia neomexicana A.Gray, AF537347; Scorpiurus vermiculatus L., AF218536; Sesbania grandiflora (L.) Poir., AF536354; Sesbania vesicaria (Jacq.) Elliott, AF398761; Syrmatium glabrum Vogel (as Lotus scoparius var. scoparius), AF218521.
