# Supplementary figures and images for: Phylogenetic Placement and Phylogeography of Large-Flowered Lotus Species (Leguminosae) Formerly Classified in Dorycnium: Evidence of Pre-Pleistocene Differentiation of Western and Eastern Intraspecific Groups
Source: Plants (Basel). 2021 Jan 28;10(2):260. doi: 10.3390/plants10020260 (PMC7911919; doi:10.3390/plants10020260)

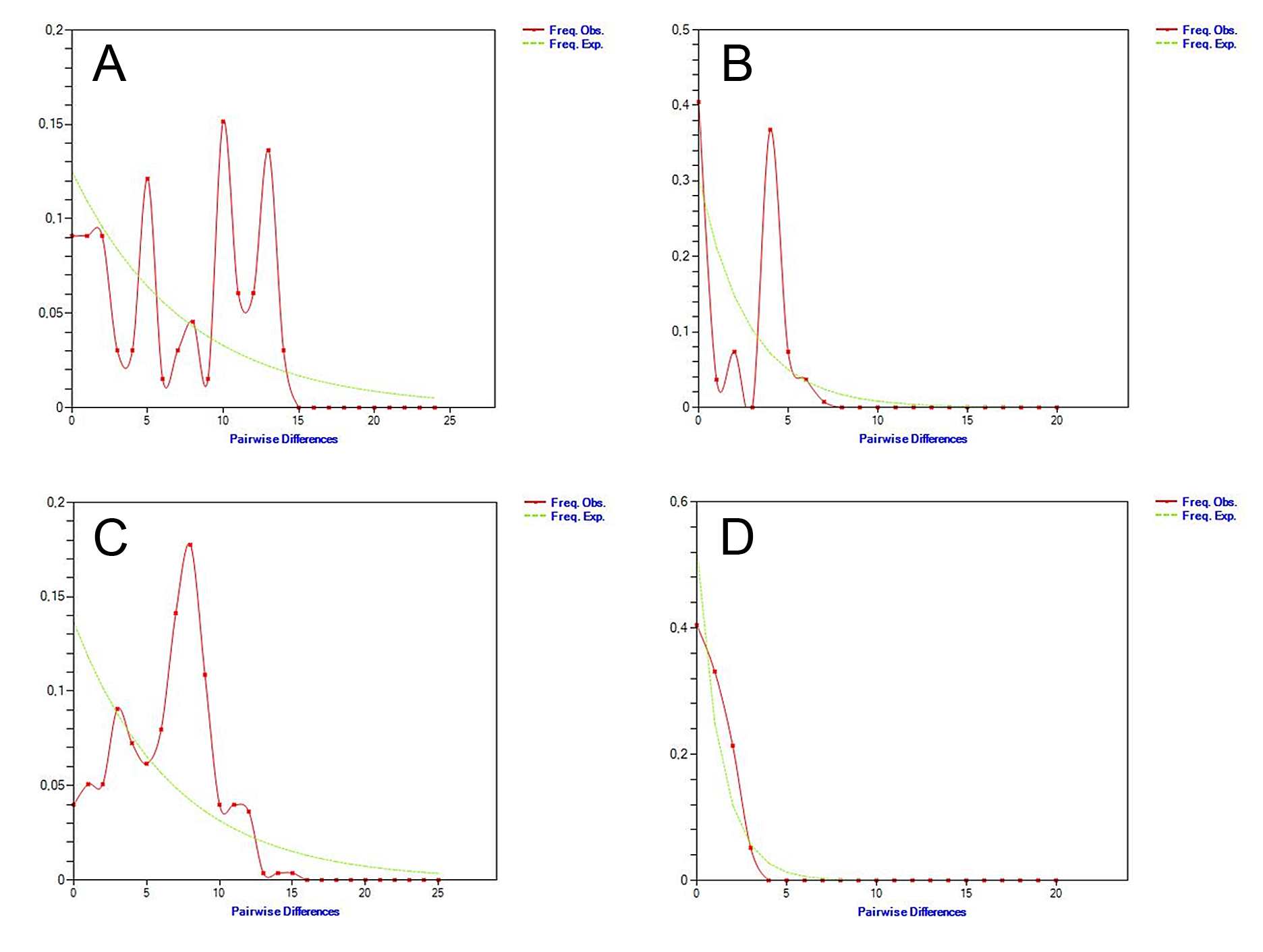

Supplement: Supplementary file 1 [file plants-10-00260-s001.zip › Figure-S1.tif]
